# Online social media tells a story of *Anaselina*, *Paraselina*, and *Selivinga* (Orthoptera, Tetrigidae), rare Australian pygmy grasshoppers

**DOI:** 10.3897/zookeys.948.52910

**Published:** 2020-07-13

**Authors:** Josip Skejo, Matthew Connors, Michael Hendriksen, Nick Lambert, Griffin Chong, Ian McMaster, Nick Monaghan, David Rentz, Reiner Richter, Kathy Rose, Damjan Franjević

**Affiliations:** 1 University of Zagreb, Faculty of Science, Department of Biology, Evolution Lab, HR-10000 Zagreb, Croatia; 2 Heinrich – Heine University, Institute for Molecular Evolution, D-40225, Düsseldorf, Germany; 3 James Cook University, Douglas, Queensland, 4811, Australia; 4 8 Belbowrie Road, Toormina, New South Wales, 2452, Australia; 5 citizen scientist (minor), Brisbane, Queensland, 4104, Australia; 6 Mount Mellum, Queensland, 4550, Australia; 7 Life Unseen, Melbourne, Victoria, Australia; 8 19 Butler Dr Kuranda, Queensland, 4881, Australia; 9 PO Box 37, Monbulk, Victoria, 3793, Australia; 10 968 Wilsons Creek Road Mullumbimby, New South Wales, 2482, Australia

**Keywords:** barkhopper, Batrachideinae, citizen science, Cladonotinae, Flickr, iNaturalist, New South Wales, Queensland, relics, Tetrigoidea

## Abstract

Knowledge on the pygmy grasshoppers of Australia is, despite the numerous endemics being described from this unique continent, still scarce. Of interest is the *Vingselina* genus group, including genera *Anaselina* Storozhenko, 2019, *Paraselina* Storozhenko, 2019, *Selivinga* Storozhenko, 2019 and *Vingselina* Sjöstedt, 1921. The systematic position of this group, currently assigned to Batrachideinae (Bufonidini), is probably not correct. In this study new records are presented of *Anaselina minor* (Sjöstedt, 1921), *Paraselina brunneri* (Bolívar, 1887), *P. trituberculata* (Sjöstedt, 1932), and *Selivinga tribulata* Storozhenko, 2019, all except *A. minor* the first records of the species since their original descriptions. The first photographs of living specimens of *A. minor*, *P. brunneri*, *P. trituberculata* and *S. tribulata* are provided and their habitats described. All the records were compiled by citizen scientists who use online social media, such as iNaturalist. Lastly, *P. multifora* (Rehn, 1952) **syn. nov**. represents a junior synonym of *P. brunneri*.

## Introduction

A long history of existence in isolation has resulted in Australia being one of the strongholds of Earth’s biodiversity. It is a place where more than 80% of fauna and flora are endemic (Egerton and Lochman 2009, Williams et al. 2011), such as monotremes (Monotremata), numerous marsupials (Marsupialia), emus (*Dromaius novaehollandiae*), lyrebirds (e.g., *Menura alberti*), or the Wollemi pine (*Wollemia nobilis*) (Waisbecker and Beck 2015; Jones et al. 1995). Especially interesting are eastern rainforests, which harbor great number of endemics and relics, and should be considered a world biodiversity hotspot (Williams et al. 2011). Those rainforests are inhabited by Australian barkhoppers and helmed groundhoppers, small pygmy grasshoppers (Orthoptera: Tetrigidae: genera *Anaselina*, *Paraselina*, *Selivinga*, *Vingselina*) found on bark, and among the rarest grasshoppers on the planet from the point of view of the numbers of records (Bolívar 1887; Sjöstedt 1921, 1932; Rehn 1952; Storozhenko 2019). The Australian barkhoppers and helmed groundhoppers are currently assigned to the subfamily Batrachideinae and the tribe Bufonidini, together with *Vilma* from the Solomon Islands, *Hyperyboella* from New Caledonia, and *Bufonides* from New Guinea (Storozhenko 2019; Skejo et al. 2020). Previous authors included those genera in Cladonotinae (Rehn 1952) and where they really belong remains an open question. Altogether, six species are known, chronologically sorted by the time of discovery: 1) *Paraselina brunneri* (Bolívar, 1887), 2) *Vingselina crassa* Sjöstedt, 1921, 3) *Anaselina minor* (Sjöstedt, 1921), 4) *P. trituberculata* (Sjöstedt, 1932), 5) *P. multifora* (Rehn, 1952), 6) *Selivinga tribulata* Storozhenko, 2019 (Bolívar 1887; Sjöstedt 1921, 1932; Rehn 1952; Storozhenko 2019). Sjöstedt (1921) defined the genus *Vingselina*, while Storozhenko (2019) defined *Anaselina*, *Paraselina*, and *Selivinga*.

**Figure 1.**
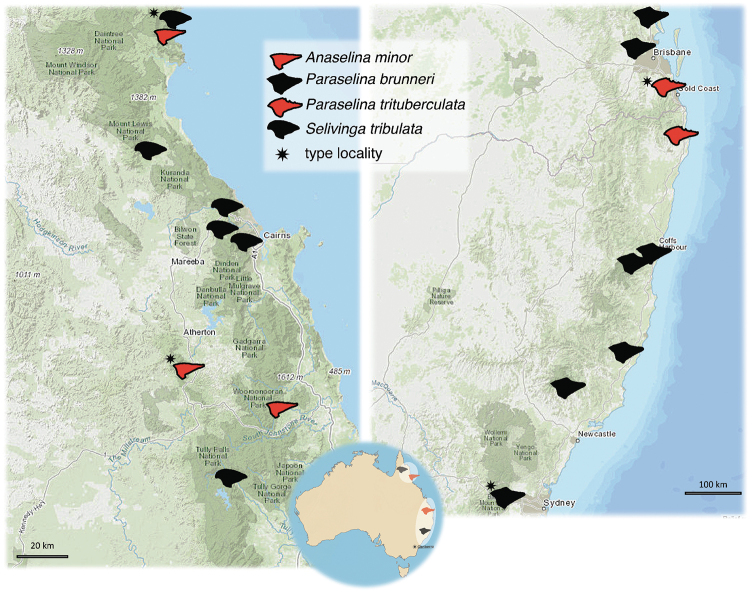
Updated distribution map of the Australian pygmy grasshoppers – *Anaselina minor*, *Paraselina brunneri*, *P. trituberculata*, and *Selivinga tribulata*. Each species is represented by its unique symbol, the silhouette of the species pronotum. The small map of Australia shows two regions inhabited by the barkhoppers.

In this study, we present eleven new records of Australian tetrigids that exhibit characters between Batrachideinae and Cladonotinae (Rehn 1952; Storozhenko 2019). Namely, we present records of *Paraselina brunneri* from iNaturalist, *P. trituberculata* from Flickr, *A. minor* from the field, and *Selivinga tribulata* from Flickr, one blog, and the field (Figure 1). These are the first findings of those species since their original descriptions, except in the case of *A. minor*. Also, we propose synonymy of *Paraselina multifora* syn. nov. with *P. brunneri*. With this paper, we show that online social media platforms are a modern tool for studying unreachable biodiversity.

## Materials and methods

### Taxonomy and nomenclature

Taxonomy follows Storozhenko’s (2019) division of the subfamily Batrachideinae into Batrachideini, Bufonidini, and Cassitettigini. Australian Batrachideinae (genera *Anaselina* – 1 sp., *Paraselina* – 2 spp. after our study, *Selivinga* – 1 sp., and *Vingselina* – 1 sp.) belong to Bufonidini, together with *Bufonides* (New Guinea), *Hyperyboella* (New Caledonia), and *Vilma* (Solomon Islands) (Steinmann 1973; Storozhenko 2019; Skejo et al. 2020; Cigliano et al. 2020). Nomenclature is in accordance with the International Code of Zoological Nomenclature (ICZN 1999).

### Morphological terminology

We follow the morphological terminology presented by Tumbrinck (2014a, 2014b, 2015) and Storozhenko (2019). All the important morphological characters used to distinguish members of the group are related to the pronotum: 1) the shape and height of the median carina of the pronotum, 2) the shape of the frontomedial projection of the pronotum, and 3) the fashion of the pronotal ending.

### Museum abbreviations

We have examined type specimens of all the Australian Batrachideinae: Bufonidini, and those specimens are the only published specimens of said species. The exception are a few non-type specimens of *Anaselina minor*, originating from Cape Tribulation, deposited in the Zoological Institute of the Russian Academy of Sciences (**ZIN)** and depicted by Storozhenko (2019). In the following museums, the type materials of the here-reported Australian Batrachideinae have been deposited:

**MCZ US** Museum of Comparative Zoology of Harvard University in Cambridge, Massachusetts, USA (types of *Vingselina multifora* Rehn, 1952);

**NHMW**Naturhistorisches Museum Wien, Wien, Austria (types of *Diotarus brunneri* Bolívar, 1887);

**NHRS**Naturhistoriska riksmuseet, Stockholm, Sweden (types of *Vingselina minor* Sjöstedt, 1921, and types of *Vingselina crassa* Sjöstedt);

**QM**Queensland Museum, Queensland, Brisbane, Australia (types of *Vingselina trituberculata* Sjöstedt, 1932);

**ZIN** Zoological Institute of the Russian Academy of Sciences, St. Petersburg, Russia (types of *Selivinga tribulata* Storozhenko, 2019).

### Online social media

Eight new records were posted on online social media by citizen scientists and then identified by specialists: iNaturalist (Table 1: records 5, 6, 9, 10, 16, Table 2), Flickr (Table 1: records 12, 14, Table 2), and the NM’s blog (Table 2: record 15). All photographs are reproduced here. Besides new records from online social media, MC observed *A. minor* in one and *S. tribulata* in two additional localities (Table 1: records 17, 18).

**Table 1.** All the known records of *Anaselina minor*, *Paraselina brunneri* (= *Paraselina multifora*), *Paraselina trituberculata*, and *Selivinga tribulata*, the Australian endemic Batrachideinae/Cladonotinae (*P. tritub.* – *Paraselina trituberculata*, NSW – New South Wales, QLD – Queensland, HT – holotype, LT – lectotype, PT – paratype, *– new records).

	Locality	Coordinates	Date	Specimen data	Reference	N
***A. minor***	QLD: Herberton	(17.38S, 145.42E)	early 1900s	1♀HT, Mjöberg, NHRS	Sjöstedt 1921	1
	QLD: Cape Tribulation tropical rainforest	16.117S, 145.433E	10.–30.III.2000.	2 ♂♂ + 1♀, S. Storozhenko (ZIN)	Storozhenko 2019	2
	QLD: Wooroonooran	17.653S, 145.718E	05.X.2019.	1♂, M. Connors	This study*	3
***P. brunneri***	NSW: Sydney	(33.68S, 150.56E)	1860s	1♀ LT, Frauenfeld, NHMW	Bolívar 1887	4
	NSW: Lansdowne Forest: Starrs Creek	31.6997S, 152.5129E	07.IV.2019.	1♀, R. Richter	iNaturalist*	5
	NSW: Upper Orara	30.2801S, 152.9441E	29.I.2019.	1♀, N. Lambert	iNaturalist*	6
	NSW: Dorrigo: 900 m a.s.l. (Macleay Range)	(30.40S, 152.65E)	1930s	1♀ HT; 2♀♀PTs, Darlington, MCZ US	Rehn 1952: *P. multifora*	7
	NSW: Salisbury	(32.19S, 151.56E)	12.II.1932.	1♀ PT, MCZ US		8
	QLD: Mount Mellum	26.8239S, 152.9199E	29.I.2019.	1 indiv., I. McMaster	iNaturalist*	9
	QLD: Mount Glorious	27.3161S, 152.7308E	02.I.2019.	1♂, G. Chong	QuestaGame, iNaturalist*	10
***P. tritub.***	QLD: Mount Tambourine	(27.88S, 153.18E)	28.X.1912.	1♀ HT, Brisbane	Sjöstedt 1932	11
	NSW: Wilsons Creek	28.5713S, 153.4265E	30.I.2013.	1♂, K. Rose	Flickr*	12
***S. tribulata***	QLD: Cape Tribulation	16.1166S, 145.4333E	10.–30.III.2000.	1♀ HT; 2♀♀, 1♂ PTs, author, ZIN	Storozhenko, 2019	13
	QLD: Kuranda	16.8050S, 145.6385E	10.XI.2010.	D. Rentz	Flickr*	14
	QLD: Julatten: Kingfisher Park	16.5940S, 145.3399E	15.X.2013.	N. Monaghan	LifeUnseen*	15
	QLD: Cardwell: Tully Gorge	17.7749S, 145.6504E	15.XI.2017.	M. Connors	iNaturalist*	16
	QLD: Speewah Conservation Park	16.88S, 145.64E	05.II.2019.	M. Connors	This study*	17
	QLD: Redlynch	16.889S, 145.686E	14.IV.2019.	M. Connors	This study*	18

**Table 2.** Australian Batrachideinae of the genera *Paraselina* and *Selivinga* in online social media. The number from the table accompanies the number of the record from Table 1.

Species	Observer	Link to the observation(s)	N
*Paraselina brunneri*	Reiner Richter	https://www.inaturalist.org/observations/22360480	5
	Nick Lambert	https://www.inaturalist.org/observations/19947697	6
	Ian McMaster	https://www.inaturalist.org/observations/35283526	7
	Griffin Chong	https://www.inaturalist.org/observations/19373510	10
*P. trituberculata*	Kathy Rose	https://www.flickr.com/photos/imbala/8466615980	11
*Selivinga tribulata*	David Rentz	https://www.flickr.com/photos/naturenoises/7570494412	14
	Nick Monaghan	https://www.lifeunseen.smugmug.com/insects/grasshoppers-crickets-katydids/grasshoppers-suborder-caelifer/tetrigidae-pygmy-grasshoppers	15
	Matthew Connors	https://www.inaturalist.org/observations/37612204	16

## Results

### New records of *Anaselina*, *Paraselina*, and *Selivinga*

In addition to the published specimens, which are mostly the type specimens’ records deposited in museum collections (see Storozhenko 2019), we present eleven new records (Table 1.) of these rare Australian pygmy grasshoppers, *Anaselina minor*, *Selivinga tribulata*, *Paraselina brunneri*, and *P. trituberculata*. All the records, except the one of *A. minor*, are the first records since the species descriptions. All known species belonging to the aforementioned genera are flightless and endemic. New and old records are summarized in Table 1 and depicted in the map (Figure 1).

**1. *Anaselina minor* (Sjöstedt, 1921), Tiny helmed groundhopper**

**Figure 2.**
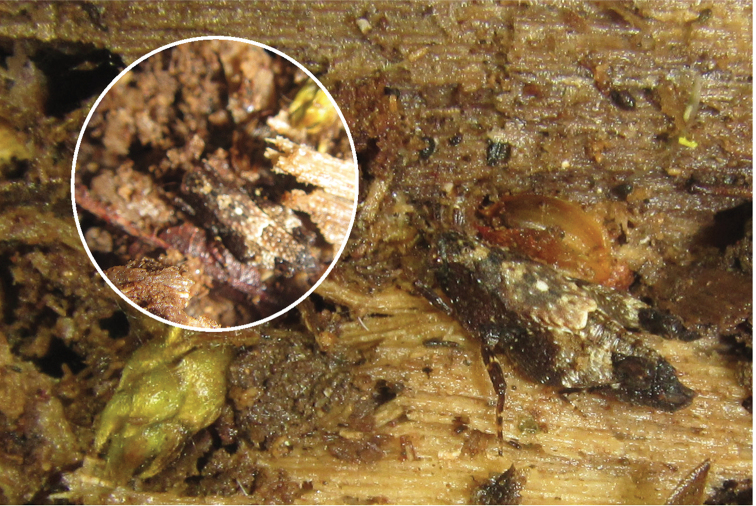
The Tiny helmed groundhopper, *Anaselina minor*, a male in the natural habitat in Wooroonooran (Matthew Connors). Note the uncovered part of the abdomen and bilobate apex of the flat pronotum.

The Tiny helmed groundhopper (Figure 2) was described under the name *Vingselina minor* one century ago (Sjöstedt 1921), based on a single female from Herberton, which lacks hind legs. Storozhenko (2019) re-described the species based on freshly collected individuals from Cape Tribulation. The species is readily distinguished from other Australian taxa by the set of the following characters: 1) flat pronotum, whose anterior margin only partly covers the fastigium; 2) short pronotum, leaving most of the abdomen uncovered; and 3) prominent fastigium (in lateral view).

The species has hitherto been known from two localities, the type locality (Sjöstedt 1921) and the Cape Tribulation (Storozhenko 2019). Here we also report a third locality, Wooroonooran, ca. 30 km NW of the type locality. The species inhabits rainforests, where it can be found in wet, but also in relatively dry leaf litter (Figure 2).

**2. *Paraselina brunneri* (Bolívar, 1887), Angled Australian barkhopper**

**Figure 3.**
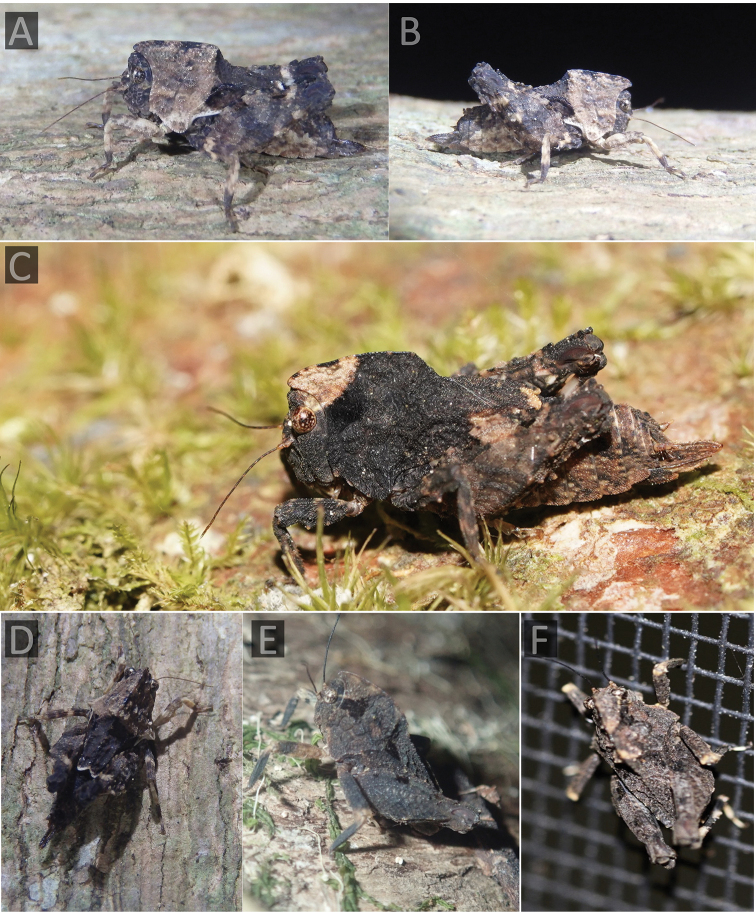
The Angled Australian barkhopper, *Paraselina brunneri* (= *P. multifora* syn. nov.). **A, B, D** a female from Upper Orara (Nick Lambert) **C** a female from Lansdowne forest (Reiner Richter) **E** a male from Mt. Glorious (Griffin Chong) **F** individual from Mt. Mellum (Ian McMaster).

The Angled Australian barkhopper *Paraselina brunneri* (Figure 3) was hitherto only known to us from a single female holotype, collected in the surroundings of Sydney, and described more than 130 years ago by Bolívar (1887). The species has never been recorded since.

Rehn (1952), when describing *P. multifora* (as *Vingselina multifora*) based on a female holotype and three female paratypes, from Dorrigo (Macleay Range) and Salisbury, did not see Bolívar’s type, but only Sjöstedt’s (1921) drawings with which he made the comparisons.

Now, after examination of the holotype of *P. brunneri* (see Cigliano et al. 2020), compared with types of *P. multifora* (see Rehn 1952) as well as with four new records (Table 1), the conclusion is clear that *P. multifora* represents a synonym of *P. brunneri*. The species inhabits rainforests of SE Queensland and NE New South Wales (Figure 1), where it dwells on bark. Griffin Chong noticed a specimen of this unusual grasshopper (Figure 3E) as an irregular bump on the bark of a tree he was walking past on the Morelia track (Mt Glorious).

Rehn (1952) described *P. multifora* but was unable to examine the holotype of *P. brunneri*. Our new records belong to the same species as Rehn’s (1952) four type specimens, and the holotype of *P. brunneri*. This species is thus known from nine specimens originating from seven localities (Table 1) and its valid name is *Paraselina brunneri*. Hence *Vingselina multifora* Rehn, 1952 syn. nov. represents a junior subjective synonym of *Paraselina brunneri*.

There is considerable infraspecific variability in size, coloration, and angulation of the median carina of the pronotum. Differences separating *P. multifora* from *P. brunneri*, reported by Rehn (1952) and based on Sjöstedt’s figure are not reliable. Those characters were 1) the prominence of the compound eyes (same in all specimens), 2) the angle of the median carina of the pronotum (reported to be more angular in *P. multifora*, but when looking at the types, the situation is opposite, with the type of *P. brunneri* being more angled), and 3) the size of hind femora (length of hind femora of *P. brunneri* holotype are 6.5 mm, while in *P. multifora* they measure 4.8–5.8 mm).

The types of the two taxa are very similar, but in addition we have new records, which provide evidence of this infraspecific variability, especially in the size and morphology of the median carina of the pronotum.

**3. *Paraselina trituberculata* (Sjöstedt, 1932), Triple-bump Australian barkhopper**

**Figure 4.**
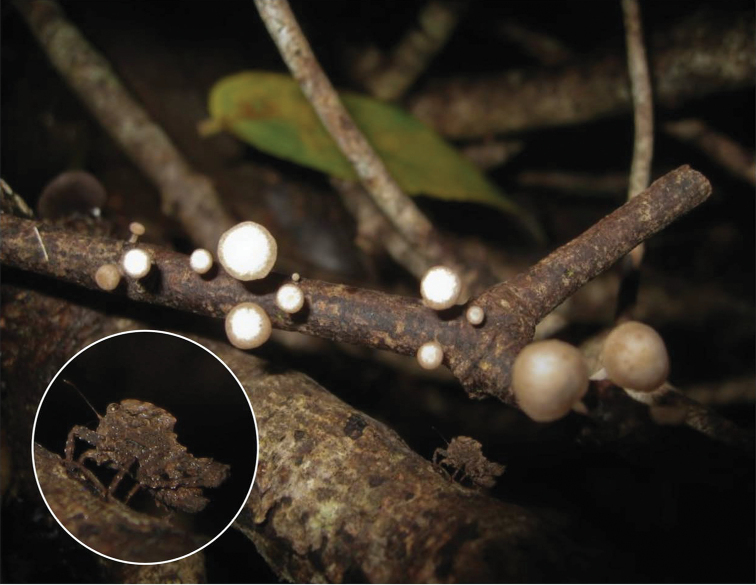
The Triple-bump Australian barkhopper, *Paraselina trituberculata* (Sjöstedt, 1932), a male in his habitat in Wilsons Creek (Kathy Rose).

The Triple-bump Australian barkhopper *Paraselina trituberculata* (Figure 4) was described almost 90 years ago (Sjöstedt 1932) from a single female (holotype) from the rainforest of the Mount Tamborine, and has never been reported again.

Here, we not only report the species for the first time since the description, but also report a male specimen for the first time. The specimen was photographed by Kathy Rose and uploaded to Flickr, where it was accidentally discovered by Josip Skejo and with help of Josef Tumbrinck identified as *Paraselina trituberculata*. The male was found in the rainforest of Wilsons Creek sitting on bark that was full of mushrooms (Figure 4, Table 1). Wilsons Creek is ca. 70 km SE from the Mount Tamborine, the type locality of the species.

The species is known from two rainforest localities (Figure 1) situated on the border of Queensland and New South Wales, south of Brisbane. *P. trituberculata* is easily distinguished from Australian Batrachideinae and all the other pygmy grasshoppers in the world by the angled pronotum (high in the cephalic part, low in caudal part) that bears three smaller warts on the median carina (Figure 4).

**4. *Selivinga tribulata* Storozhenko, 2019, Tribulation helmed groundhopper**

**Figure 5.**
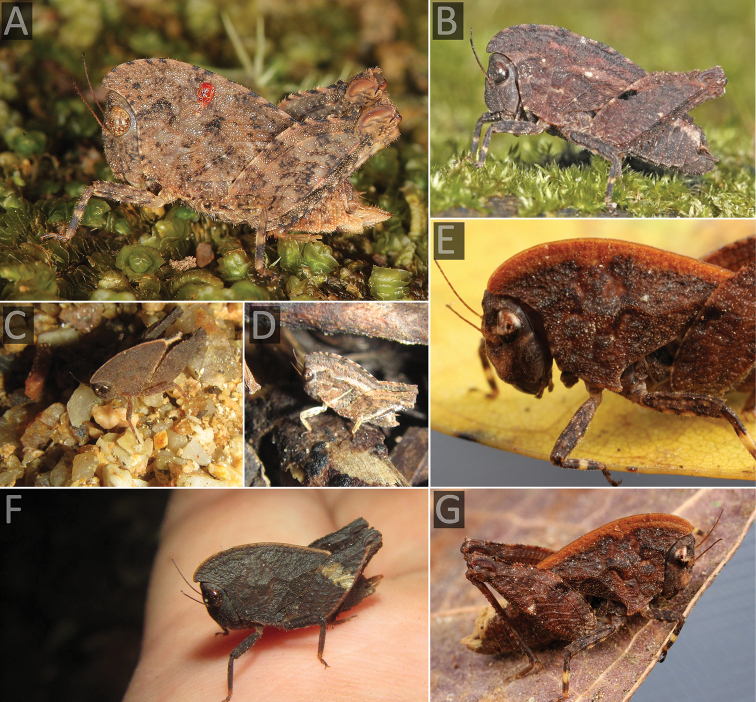
The Tribulation helmed groundhopper, *Selivinga tribulata*, Living specimens in natural habitat. **A** Female from Kuranda (David Rentz) **B** male from Kuranda (David Rentz), male from Tully Range (Matthew Connors) **D** nymph from Redlynch (Matthew Connors) **E, G** a male from Kingfisher park (Nick Monaghan) **F** female from Speewah (Matthew Connors).

The Tribulation helmed groundhopper (Figure 5) was described under the name *Selivinga tribulata* in 2019 by Storozhenko. This is the first crested Australian species to be described and one of the most easily recognizable species from the continent. Not only is it easily identifiable by its prominent crest, but also by the low position of the antennal grooves.

**Figure 6.**
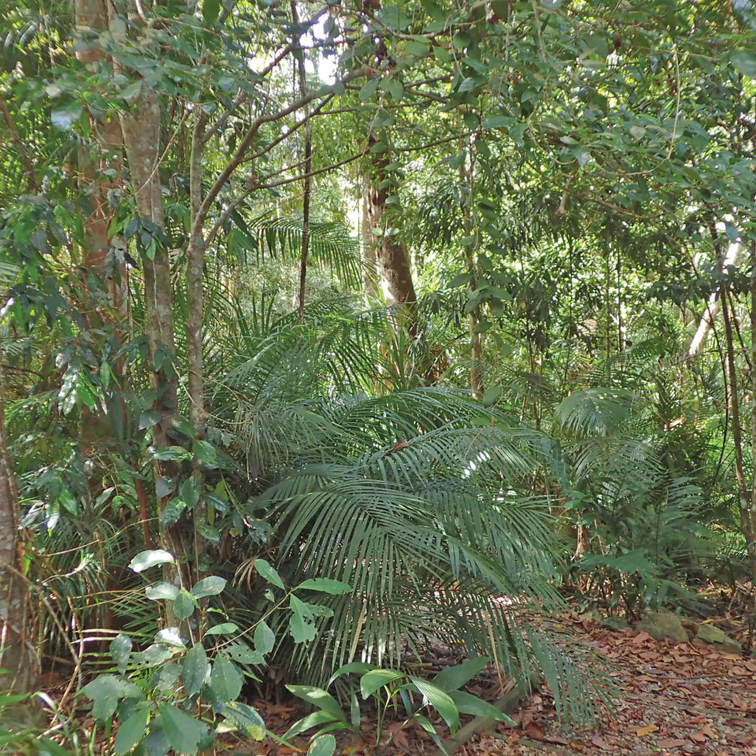
Habitat of the Tribulation helmed groundhopper, *Seliving tribulata*. Rainforest in Kuranda, Queensland, rich in dry leaflitter (David Rentz).

The species inhabits the northern part of Queensland and is, for now, known from Cape Tribulation (the type locality), Kuranda (Figure 6), Tully Gorge, and Kingfisher Park (see Figure 1). *Selivinga tribulata* is a rainforest species. It can be found in relatively dry leaf litter as well as the normally moist leaf litter that one associates with rainforests, but also is attracted to light. In Kuranda (Queensland) adults have been observed every month of the year.

Even though the species has been described only recently, DR had already been aware of its existence since 2000, and there is indeed a notable population in the author’s garden (see habitat in Figure 6). Specimens were observed in every month of the year. JS found photos of the species a few years ago in Flickr and fortunately, in 2019, the name for the species became available.

## Discussion

Even though throughout the study we call them Batrachideinae, and even though Australian barkhoppers and helmed groundhoppers were recently placed in the tribe Bufonidini, together with New Guinean *Bufonides*, it remains questionable as to where *Anaselina*, *Paraselina*, *Selivinga*, *Vingselina*, and *Vilma* really belong. Rehn (1952) placed those genera under Cladonotinae and noted that they should not be assigned to Batrachideinae, as they bear similarities with *Diotarus* and *Piezotettix*, which are true Cladonotinae. They were assigned to Batrachideinae, with no later discussion as to whether they should be (Storozhenko 2019). *Bufonides*, after which the Bufonidini obtained their name, has a rather different morphology: it has antennae with more than 20 segments (Hinton 1940), a typical Batrachideinae character, contrary to less than 18 in the Australian genera, so it is questionable that they belong to the same group as *Bufonides*. It is clear that systematics based on morphological characters alone has limitations, and new studies based on molecular analysis are therefore needed. It is also clear that the current subfamilies that are recognized since 1887 (Bolívar 1887), are in need of revision, as many of these are clearly polyphyletic or paraphyletic taxa (e.g., Metrodorinae and Cladonotinae) (pers. comm. H. Devriese).

Poor taxonomic knowledge does not prevent us from assessing distributions of Australian barkhoppers, nor from separating species. Species of the genera *Anaselina*, *Paraselina*, and *Selivinga* inhabit humid forests of eastern Australia and are easily distinguishable from each other. In the past it was difficult to study material from different places rapidly. In this study, we show that mainly with records from online social media platforms we can add knowledge to the biology and taxonomy of certain species. Today, it is much easier to study material from all over the world and communicate with experts and citizen scientists. Citizen science records contributed to the knowledge of morphology of *Paraselina brunneri* and *P. multifora*, which had not been recorded for many decades. From these photos, we have found that specimens vary in certain morphological traits and that micro-differences used to separate *P. brunneri* from *P. multifora* are not species-specific, so *P. multifora* should be considered a synonym of *P. brunneri*.

*Anaselina minor* is the smallest member of the *Vingselina* genus group, now known from three localities in the northern Queensland. *Selivinga tribulata* was described only last year from Cape Tribulation and here we report three new localities for this species, with a description and depiction of its habitat. We also present the very first record of *Paraselina trituberculata* since its description, and confirm that the species is only overlooked, not extinct. Social media platforms are already used by scientists, and studies which will make it available for people to record and learn about species in a more visual and simple manner, are strongly needed.

In conclusion, *A. minor*, *P. brunneri*, *P. trituberculata*, and *S. tribulata* are easily identifiable, but rare species. Most of the knowledge on their biology was hitherto based on old museum specimens. Here, with united forces of citizens, who post photos online, and experts, who use online social platforms in order to identify specimens, we present an annotated distribution map of the aforementioned species, as well as a taxonomic scrutiny on the system of their classification. Citizen science is not age-limited nor profession-limited. In 2020, anybody and everybody can contribute to biodiversity studies. Evidence is presented by the authors of this study, among which there are an 11-year-old boy and a retired English teacher.
